# A gluten-free diet effectively reduces symptoms and health care consumption in a Swedish celiac disease population

**DOI:** 10.1186/1471-230X-12-125

**Published:** 2012-09-17

**Authors:** Fredrik Norström, Olof Sandström, Lars Lindholm, Anneli Ivarsson

**Affiliations:** 1 Department of Public Health and Clinical Medicine, Epidemiology and Global Health, Umeå University, Umeå, Sweden; 2 Department of Clinical Sciences, Pediatrics, Umeå University, Umeå, Sweden

## Abstract

**Background:**

A gluten-free diet is the only available treatment for celiac disease. Our aim was to investigate the effect of a gluten-free diet on celiac disease related symptoms, health care consumption, and the risk of developing associated immune-mediated diseases.

**Methods:**

A questionnaire was sent to 1,560 randomly selected members of the Swedish Society for Coeliacs, divided into equal-sized age- and sex strata; 1,031 (66%) responded. Self-reported symptoms, health care consumption (measured by health care visits and hospitalization days), and missed working days were reported both for the year prior to diagnosis (normal diet) and the year prior to receiving the questionnaire while undergoing treatment with a gluten-free diet. Associated immune-mediated diseases (diabetes mellitus type 1, rheumatic disease, thyroid disease, vitiligo, alopecia areata and inflammatory bowel disease) were self-reported including the year of diagnosis.

**Results:**

All investigated symptoms except joint pain improved after diagnosis and initiated gluten-free diet. Both health care consumption and missed working days decreased. Associated immune-mediated diseases were diagnosed equally often before and after celiac disease diagnosis.

**Conclusions:**

Initiated treatment with a gluten-free diet improves the situation for celiac disease patients in terms of reduced symptoms and health care consumption. An earlier celiac disease diagnosis is therefore of great importance.

## Background

In celiac disease (CD), gluten triggers an autoimmune reaction in the small intestinal mucosa which results in inflammation, villous atrophy, and malabsorption. A gluten-free diet is the only effective treatment for CD, and it usually results in recovery of the small intestinal mucosa [1].

During the last 30 years the clinical spectrum of CD has changed from mainly being comprised of young children with overt malabsorption to often affecting adults, with mild or atypical symptoms, and some of those diagnosed with the disease are even asymptomatic [2]. Despite an increased awareness of symptoms related to CD, the delay from the first appearance of CD related symptoms to diagnosis is still long [3-5]. Economic consequences of CD for individuals and society have undergone little study. It has been shown, however, that the cost of food for CD patients is higher than for non-CD persons [6], and that women with CD consume more health care than other women [7]. CD diagnosis and treatment has also been shown to decrease the costs for medical care in the United States, suggesting that a diagnosis can convey economic savings for society [8,9]. There is an association between CD and other immune-mediated diseases, with CD being more prevalent among diabetes mellitus type 1 and thyroid disease patients [10-12] and inflammatory bowel disease and thyroid disease being more prevalent among CD patients [13-15]. A protective effect of a gluten-free diet on the risk of developing these related diseases has been proposed [16,17], but there is uncertainty in this regard [15,18-20].

The aim of this study was to investigate the effect of a gluten-free diet on CD related symptoms, health care consumption, and the risk of developing associated immune-mediated diseases.

## Methods

### Study design

A cross-sectional questionnaire survey among Swedish adults with CD was performed during 2009 [5]. The questionnaire was approved by the Regional Ethical Review Board at Umeå University and an English translation is available online at Biomed Central [21].

### Subjects

We invited randomly selected members of the Swedish Society for Coeliacs to respond to a postal questionnaire administered by the Society, and when needed three reminders were sent. The Society represents about 60% of Swedish CD patients [5]. A questionnaire was sent to 65 males and 65 females with reported CD in five-year age intervals from 20 years of age and above (20–24, 25–29, …, 70–74, and 75 years or older), totaling 1,560 members. There were 1,031 (66%) eligible responses to the questionnaire. Members self-report their CD diagnosis when joining the Society. As the diagnosis is not verified by the Society, we used information from the questionnaire about how members were diagnosed (blood sample, biopsy, and/or diet change) and if they were recommended adherence to a gluten-free diet by a medical professional. We excluded 91 questionnaires where either a CD diagnosis could not be verified or age and/or sex were not consistent with information in the member register of the society [5]. We defined 288 responders as diagnosed due to screening of CD risk groups. For them the primary investigation for CD was based on a disease with a known relationship to CD or due to heredity for CD. Among responders, 96% reported a strict compliance with a gluten-free diet, 52% (n = 536) were women, and the mean age was 52 years (Table 1). More characteristics of the study population, as well as more details about inclusion criteria, are available in a previous publication from the questionnaire study [5]. Questionnaires were scanned and checked for consistency.

**Table 1:** Characteristics of celiac disease subjects

	**n**	**%**	**Mean**	**Median**	**Quartile**	
					**1st**	**3rd**
Participants	1,031					
Males/Females	495/536	48/52				
Age when responding (years)	1,031		52	53	36	67
Age at diagnosis (years)	945		39	41	27	53
Duration of celiac disease diagnosis (years)^a^	945		10	13	5	19
Compliance with a gluten-free diet	1,025					
Strict/Non-strict	979/46	96/4				

^a^ Members were invited based on entrance to society latest 1^st^ of April 2009. Duration of celiac disease diagnosis was calculated as years ahead of 2009 that respondent was diagnosed.

### The questionnaire

**Figure 1:**
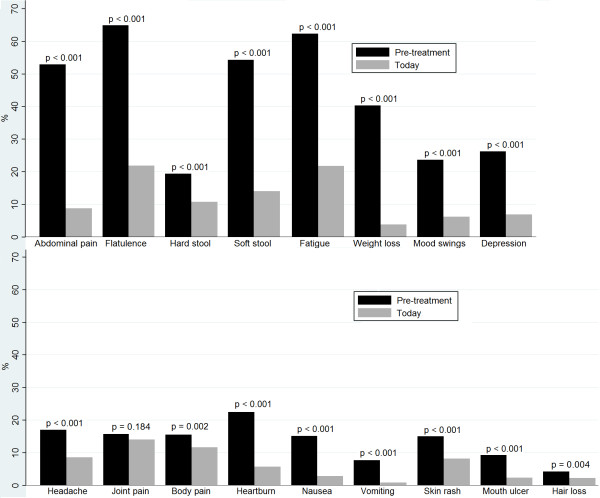
**Proportion of celiac disease patients reporting major (“often” or “always”) symptoms the year prior to celiac disease diagnosis (pre-treatment) and after initiated gluten-free diet (today).** Number of respondents for each symptom ranged between 879 and 949. Differences in symptoms *pre-treatment* and *today* were tested with the sign test.

The questionnaire included sections covering self-reported symptoms, health care consumption, missed working days, and self-reported diseases. For each symptom (listed in Figure 1) there were five possible answers, which were dichotomized to major (”often” and ”always”) and minor severity (“never”, ”rarely”, and ”sometimes”). Health care consumption included number of health care visits, hospitalization days, and drug use. Missed working days also included missed school days and similar circumstances. For both symptoms, health care consumption, and missed working days the respondents were asked about the situation both the year prior to initiated treatment for CD, referred to as *pre-treatment*, as well as the year prior to responding to the questionnaire, referred to as *today*. Regarding drug use, respondents were asked if their CD diagnosis and gluten-free diet had resulted in them being able to stop taking any medication.

Respondents reported whether they had the following immune-mediated diseases: diabetes (both insulin and non-insulin dependent; the latter is not autoimmune in nature), rheumatic disease, thyroid disease, vitiligo, alopecia areata, or inflammatory bowel disease. Regarding these diseases the respondents were also asked for the year of diagnosis. As respondents were asked about insulin or non-insulin dependent diabetes, we cannot with certainty differentiate between diabetes mellitus type 1 and 2.

### Statistical analysis

Results are presented using descriptive statistics. The sign-test was used for comparisons between *pre-treatment* and *today*. Time between diagnoses was calculated as time from CD diagnosis to other disease diagnosis. When both diagnoses occurred during the same year, time was defined as 0 years. Differences in symptoms between members with a recent diagnosis (2005–2009) and members with earlier diagnosis were performed using Students *t*-test. Comparisons of health care visits, hospitalization days, and missed working days between the groups were performed using Wilcoxon rank-sum test. To test if a gluten-free diet might decrease the risk for associated diseases, the proportions of diagnoses of associated diseases before or during the same year as the CD diagnosis were compared with the proportions after CD diagnosis using Students *t*-test. We excluded comparisons for vitiligo and alopecia areata due to too few complete answers. Statistical significance was defined at the 5% level. Microsoft Access was used for data handling, while Stata 11.2 (StataCorp LP, College Station, TX) was used for statistical analysis and figures.

## Results

### Symptoms

*Pre-treatment,* flatulence (64%) was the most commonly reported symptom, followed by fatigue (62%), soft stool (54%), and abdominal pain (53%). All investigated symptoms, except joint pain, improved after diagnosis and initiated treatment with a gluten-free diet (Figure 1). *Today*, flatulence and fatigue were also the most commonly reported symptoms for all participants, even those who did not report the symptom *pre-treatment* (Table 2). It was less common that participants without a specific symptom reported *pre-treatment* reported the symptom *today* than it was for participants who reported the symptom *pre-treatment*. There were improvements in all investigated symptoms in the screening-detected cases except joint pain and body pain. Due to too few cases, comparison between *pre-treatment* and *today* was not feasible for vomiting and hair loss in the screening-detected group. For recently diagnosed cases (2005–2009), all investigated symptoms except vomiting and hair loss, which were rare in our study population, improved after diagnosis. The only differences between recently diagnosed and those with an earlier CD diagnosis was that weight loss and vomiting were less common in the former *pre-treatment*, joint pain was more common for recently diagnosed cases *pre-treatment,* and nausea was less common for recently diagnosed cases *today*.

**Table 2:** **Patients with symptoms *today *, also presenting for patients asymptomatic *pre-treatment ***

**Symptom**	**All**		**Asymptomatic**^**a**^		
	**n**^**b**^	**%**^**b**^	**n**^**a**^	**n**^**b**^	**%**^**b**^
Abdominal pain (n = 918)	81	8.8	432	14	3.2*
Flatulence (n = 897)	189	21	324	46	14*
Hard stool (n = 879)	95	11	708	43	6.1*
Soft stool (n = 926)	130	14	423	24	5.7*
Fatigue (n = 927)	202	22	349	42	12*
Weight loss (n = 903)	35	3.8	539	11	2.0*
Mood swings (n = 903)	56	6.2	689	13	1.9*
Depression (n = 913)	63	6.9	673	13	1.9*
Headache (n = 894)	77	8.6	742	21	2.8*
Joint pain (n = 897)	126	14	756	48	6.3*
Body pain (n = 897)	105	12	758	43	5.7*
Heartburn (n = 891)	51	5.7	691	16	2.3*
Nausea (n = 898)	26	2.9	762	12	1.6*
Vomiting (n = 910)	8	0.8	840	6	0.7
Skin rash (n = 898)	74	8.2	763	26	3.4*
Mouth ulcer (n = 904)	21	2.3	820	10	1.2*
Hair loss (n = 880)	20	2.3	843	9	1.1*

^a^ Not reporting specific symptom *pre-treatment*.

^b^ Reporting symptom *today*.

* Asymptomatic patients report fewer problems with symptom than symptomatic patients *today*.

### Health care consumption

Participants reported more frequent health care visits *pre-treatment* (5.4 visits during the year) than *today* (3.7 visits during the year) (p < 0.001), more hospitalization days (2.3 days during the year) *pre-treatment* compared to *today* (0.7 days during the year) (p < 0.001), and more missed working days *pre-treatment* (7.2 days during the year) than *today* (2.5 days during the year) (p < 0.001) (Table 3). For screening-detected CD patients there were also reductions in health care visits, hospitalization days and missed working days between *pre-treatment* and *today*. For recently diagnosed patients (2005–2009) there were reductions in health care visits and missed working days, but not in hospitalization days. We observed significantly fewer hospitalization days *pre-treatment* and significantly fewer health care visits *today* for recently diagnosed patients as compared to patients earlier diagnosed. Thirteen percent of participants (n = 136) reported that they stopped taking at least one drug after their CD diagnosis.

**Table 3:** **Health care consumption *pre-treatment *and *today ***

**Disease**			**Pre-treatment**			**Today**			**p**^**a**^
		**n**	**Mean**	**Median**	**SD**	**Mean**	**Median**	**SD**	
All									
	Health care visits	814	5.4	3	7.8	3.7	2	8.0	<0.001
	Hospitalization	836	2.3	0	8.5	0.7	0	4.0	<0.001
	Missed working days^b^	754	7.2	0	16	2.5	0	9.6	<0.001
Screening-detected cases^c^									
	Health care visits	144	4.8	2	9.0	4.1	1	9.9	0.001
	Hospitalization	151	3.0	0	11	1.0	0	5.4	0.036
	Missed working days^b^	136	9.0	0	21	1.8	0	7.4	<0.001
Recent diagnosis^d^									
	Health care visits	202	5.0	3	7.1	4.3	2	8.8	<0.001
	Hospitalization	200	0.62	0	2.7	0.36	0	1.8	0.860
	Missed working days^b^	188	7.5	0	17	3.4	0	13	<0.001

^a^ Using the sign test.

^b^ Also including missed school days and similar circumstances.

^c^ Primary investigation started based on a disease with known relation to CD or due to heredity for CD.

^d^ CD diagnosis between 2005 and 2009.

### Self-reported immune-mediated diseases

**Figure 2:**
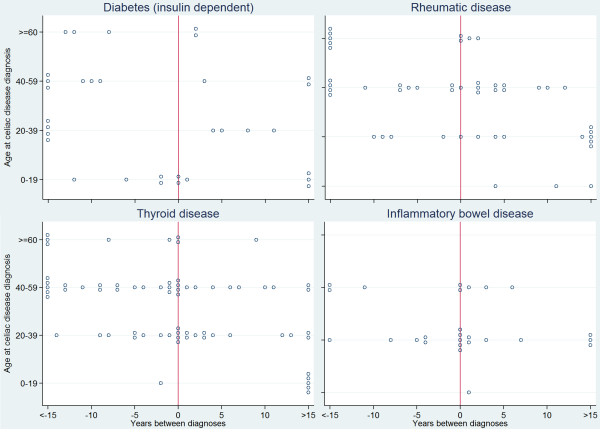
**Difference in years between the diagnosis of celiac disease and the diagnosis of associated immune-mediated disease.** Difference positive if celiac disease diagnosis first and 0 if both diagnoses during same year.

At least one immune-mediated disease was reported by 256 (25%) of the study participants. Autoimmune thyroid disease, reported by 9.1%, was the most prevalent immune-mediated disease (Table 4). There was a predominance of females for rheumatic disease and autoimmune thyroid disease but not for other self-reported associated diseases. There was no difference in the frequency of being diagnosed prior to or after CD diagnosis for any of the associated diseases (Figure 2).

**Table 4:** Proportion of immune-mediated diseases and time development in relation to celiac disease (CD) diagnosis

		**All**	**Males**	**Females**	**p**^**a**^	**Before**	**Jointly**^**b**^	**After**	**n**^**c**^	**p**^**d**^
Diabetes, insulin	*n*	39	24	15	0.19	53%	6%	41%	32	0.29
	*%*	3.8	4.8	2.8						
Diabetes, non-insulin	*n*	24	17	7	0.04	20%	13%	67%	15	0.20
	*%*	2.3	3.4	1.3						
Rheumatic disease	*n*	80	19	61	<0.01	42%	8%	50%	48	1.00
	*%*	7.8	3.8	11						
Thyroid disease	*n*	94	19	75	<0.01	47%	15%	38%	68	0.05^e^
	*%*	9.1	3.8	14						
Vitiligo	*n*	39	16	23	0.24	72%	11%	17%	18	<0.01
	*%*	3.8	3.2	4.3						
Alopecia areata	*n*	19	11	8	0.52	62%	8%	31%	13	0.16
	*%*	1.8	2.2	1.5						
Inflammatory bowel disease	*n*	44	17	27	0.09	31%	27%	42%	26	0.43
	*%*	4.3	3.4	5.0						
*Any immune-mediated disease*^*e*^	*n*	256	170	86	<0.01	n/a^f^	n/a	n/a	n/a	n/a
	*%*	25	32	17						

^a^ Comparing males with females using Students *t*-test.

^b^ CD associated immune-mediated disease reported for same year as CD diagnosis.

^c^ Specified year for both CD and other diagnosis.

^d^ Comparing proportion with auto-immune disease diagnosed before or jointly with CD with proportion after CD diagnosis.

^e^ Non-significant.

^f^ Not including non-insulin dependent diabetes.

^g^ Comparisons not applicable for “*Any disease*”.

## Discussion

CD patients suffer from more symptoms and consume more health care before diagnosis and initiated gluten-free diet than they do afterwards. However, we did not observe a difference in the risk of developing other immune-mediated diseases after initiated treatment with a gluten-free diet.

This study is one of the largest of its kind and it has a high response rate. Its unique contribution is that it compares the situation before and after initiation of a gluten-free diet, including symptoms and health care consumption, which adds valuable information about individual and societal costs of untreated CD.

Our retrospective approach involves the risk of recall bias. We observed a similar pattern for symptom relief, health care visits, and missed working days when restricting our analysis to screened and recently diagnosed (2005–2009) CD patients. There was not a significant reduction in hospitalization days for the recently diagnosed (2005–2009) CD patients. This could indicate an improved situation due to earlier diagnosis, which we have reported earlier in the same population [5], or it might be due to problems remembering hospitalization days if diagnosed earlier than 2005. In an attempt to assess if the risk of developing associated immune-mediated diseases is affected by a gluten-free diet, we assumed that if fewer were diagnosed with the related disease after the CD diagnosis this would indicate a protective effect. The patient is likely to remember which disease was diagnosed first, but if there is a delay in diagnosis for one of the diseases the association could still be incorrect. A causal relation cannot be determined with certainty by a cross-sectional questionnaire study. An examination of hospital files would have been a valuable addition in this respect, but that was not within the scope of this study.

Retrospectively reported symptoms prior to a CD diagnosis have been studied previously [4], as have symptoms at the time of CD diagnosis [22-25]. Most studies have reported symptoms at the time of diagnosis that were obtained from medical records, making comparisons with our results difficult. Our main interest was to detect experienced changes in symptoms after initiated treatment with a gluten-free diet. Previously, similar comparisons were done for a more limited number of symptoms by Murray and colleagues in the United States, showing a similar positive pattern for investigated symptoms after initiated treatment for CD [26]. Also Ukkola with colleagues showed an improvement in symptoms after initiated treatment with a gluten-free diet for Finnish CD patients [27]. Little is known about the added costs of CD for individuals and society. Although many participants had their CD for a long time, and therefore were considerably older *today*, their health consumption was significantly lower *today* than prior to the CD diagnosis, indicating a decreased need for health care after initiated treatment with a gluten-free diet.

There is a well-known association between CD and other immune-mediated diseases [1]. A protective effect of a gluten-free diet was proposed more than a decade ago [16,28], but later studies have shown conflicting results [15,18]. In our study 25% of the individuals reported associated immune-mediated diseases. The prevalences of autoimmune thyroid disease and diabetes mellitus type 1 were similar to figures previously reported in a Swedish CD population study based on patient chart reviews [25]. Our results could not verify or reject a risk reduction effect of a gluten-free diet on the development of any of the associated immune-mediated diseases that were studied. Further studies are needed to investigate this relationship.

In a previous publication based on the same study population and questionnaire, we reported that there is a long delay until CD diagnosis and that CD patients experience a poor health-related quality of life that is significantly improved after initiation of a gluten-free diet [5]. Considering this and the results of the present study, there is a strong implication that greater effort must be made to diagnose CD earlier to decrease the burden of both poorer health-related quality of life and CD-related symptoms. This would also result in economic savings for society in terms of a reduction in health care consumption and missed working days.

Recent studies have indicated that the extent of symptoms that patients detected through a population-based CD screening might have may be similar to that of non-CD persons [29,30]. The screening-detected cases in our study were mainly from risk groups. They reported the same positive effect of symptom relief after diagnosis and initiated treatment with a gluten-free diet as the CD patients who had their primary investigation due to symptoms.

## Conclusion

In conclusion, CD patients profit from being diagnosed and treated with a gluten-free diet, since this reduces both symptoms and health care consumption. An earlier celiac disease diagnosis is therefore of great importance. The possible protective role of a gluten-free diet regarding the development of other immune-mediated diseases remains to be demonstrated.

## Competing interests

The authors declare that they have no competing interests.

## Authors’ contributions

Study design by FN, AI, and LL. FN coordinated data acquisition. FN performed the analyses and the interpretation in collaboration with AI, LL, and OS. FN drafted the paper and all co-authors contributed actively. All authors read and approved the final manuscript.

## Pre-publication history

The pre-publication history for this paper can be accessed here:

http://www.biomedcentral.com/1471-230X/12/125/prepub
